# Network as a Biomarker: A Novel Network-Based Sparse Bayesian Machine for Pathway-Driven Drug Response Prediction

**DOI:** 10.3390/genes10080602

**Published:** 2019-08-09

**Authors:** Qi Liu, Louis J. Muglia, Lei Frank Huang

**Affiliations:** 1 Brain Tumor Center, Division of Experimental Hematology and Cancer Biology, Cincinnati Children’s Hospital Medical Center, Cincinnati, OH 45229, USA; 2 Department of Pediatrics, College of Medicine, University of Cincinnati, Cincinnati, OH 45229, USA; 3 Division of Human Genetics, Cincinnati Children’s Hospital Medical Center, Cincinnati, OH 45229, USA; 4 Department of Information Science, School of Mathematical Sciences and LAMA, Peking University, Beijing 100871, China

**Keywords:** network-based sparse Bayesian machine, disease-specific driver signaling network, drug sensitivity, drug resistance, cancer signaling pathway

## Abstract

With the advances in different biological networks including gene regulation, gene co-expression, protein–protein interaction networks, and advanced approaches for network reconstruction, analysis, and interpretation, it is possible to discover reliable and accurate molecular network-based biomarkers for monitoring cancer treatment. Such efforts will also pave the way toward the realization of biomarker-driven personalized medicine against cancer. Previously, we have reconstructed disease-specific driver signaling networks using multi-omics profiles and cancer signaling pathway data. In this study, we developed a network-based sparse Bayesian machine (NBSBM) approach, using previously derived disease-specific driver signaling networks to predict cancer cell responses to drugs. NBSBM made use of the information encoded in a disease-specific (differentially expressed) network to improve its prediction performance in problems with a reduced amount of training data and a very high-dimensional feature space. Sparsity in NBSBM is favored by a spike and slab prior distribution, which is combined with a Markov random field prior that encodes the network of feature dependencies. Gene features that are connected in the network are assumed to be both relevant and irrelevant to drug responses. We compared the proposed method with network-based support vector machine (NBSVM) approaches and found that the NBSBM approach could achieve much better accuracy than the other two NBSVM methods. The gene modules selected from the disease-specific driver networks for predicting drug sensitivity might be directly involved in drug sensitivity or resistance. This work provides a disease-specific network-based drug sensitivity prediction approach and can uncover the potential mechanisms of the action of drugs by selecting the most predictive sub-networks from the disease-specific network.

## 1. Introduction

It has been reported that some cancer cells are sensitive to drugs while others are not. Meanwhile, the same drug has different efficacy on different cancer cell lines. For example, among 14 lung cancer cell lines, H1666 and Cal12T are sensitive to Dasatinib [1] while the other 12 cell lines, H322, H661, H460, H1568, H226, A549, H522, H2087, H1755, H1395, HCC364, and H2405, are not. For prostate cell lines, PC3, DU145, HPV10, LNCaP, RWPE1, HPV7, NB26, PWR1E, NB11, and W99 are sensitive to Dasatinib, however, 22Rv, VcaP, MDAPCa2b, DUCap, and WPMY1 are not. These examples show that different subtypes of lung cancer cell lines and prostate cell lines exhibited different sensitivity to Dasatinib. This raises the question of whether, based on the high throughput gene expression data, we can predict the drug sensitivity of a new cancer cell. 

The question above can be considered as a typically supervised machine learning problem. A classifier can be trained based on high throughput gene expression data and the sensitivity labels of cell lines to drugs to predict drug sensitivities. In previous work, Wong [2] and Huang [3] applied basic t-test methods to find sensitive or non-sensitive biomarkers to targeted therapy and predicted the sensitivities of new cancer cell lines to the drug, according to the gene expression data. However, they only used gene expression data for classification.

It has been reported that utilizing protein–protein interaction network data as prior information can distinguish cancer patients and non-cancer patients [4,5,6,7], and is better than only using the gene expression data of cancer patients [8,9,10]. However, in high throughput gene expression data, the dimension of features d is much larger than the number of samples n, which makes it difficult to construct an optimal classifier. Combining signaling transduction pathways into a high dimensional data classification machine is a challenge. Rapaport et al. [5] used the protein–protein interaction data as a graph and made a spectral decomposition of the gene expression data according to the characteristic functions of the graph for frequency features, and then designed an SVM classifier based on the features to classify yeasts with or without light radiation. Different from extracting the network features directly, Zhu et al. [6] constructed an SVM classifier based on gene expression data directly to classify the status of Parkinson’s patients by taking network data as a punishment term. Gönen et al. [11] combined kernel-based non-linear dimensionality reduction and binary classification to build a Bayesian algorithm under a multitask learning framework, which can reduce the off-target effects and experimental noise. Moreover, Herndaniel et al. [12] and Miguel et al. [13] developed a sparse Bayesian classifier (SBC) to classify high throughput data, integrating the gene expression data with protein–protein interactions, which was different to using gene expression data to obtain SVM classifiers, and showed better results than the network-based SVM classifiers. Additionally, Yang et al. [14] raised the network-based method, NRL2DRP, which predicts drug responses not only based on PPI data but also on the similarity of cell lines, reaching relatively high performance under cross-validation on the GDSC dataset and methods comparison.

In this study, we propose a new network-based sparse Bayesian machine (NBSBM) method by combining a sparse Bayesian classifier with a Laplace graph, which is designed by a disease-related signaling network. Previously, we have developed several disease-specific driver signaling network identification approaches to identify the potential disease-driver networks by integrating the DNA-seq, copy number, RNA-seq, and methylation profiles of cancer patients [9,10,15,16]. We took advantage of these previously identified disease-specific networks and put them as prior information for drug sensitivity or resistance prediction in NBSBM. An expectation propagation strategy was employed to obtain the optimal solution of NBSBM. We then compared the performance of NBSBM with other network-based SVM classifiers. NBSBM demonstrated much better results than the other classifiers. Furthermore, the NBSBM approach is capable of selecting the most predictive networks as a biomarker for drug sensitivity or resistance prediction.

## 2. Materials and Methods

### Sparse Bayesian Classifier Combined with Disease-Specific Network

Specifically, we consider this to be a supervised machine learning problem. The training set $D={\left\{\left(x_{i},y_{i}\right)\right\}}_{i=1}^{n}$ has features $x_{i}\in {\mathbb{R}}^{d+1}$ of which the zero-th component is equal to 1 and $x_{i}$ contains information about the gene expression or transcriptional response of cancer cells. On the other hand, $y_{i}\in \left\{-1,1\right\}$ is the class labels representing the phenotype data of the cancer cell response to drugs, while 1 represents “sensitive” and −1 represents “resistant”. We aimed to build an optimal linear classifier $\beta =\left(\beta_{0},\beta_{1},\cdots ,\beta_{d}\right)$ that utilizes a specific cancer signaling network as prior information and maximizes the distance between those sensitive and non-sensitive samples. Herbrich et al. [17] considered the existence of a true classifier $\beta_{true}$, which was used to label the data according to the rule $y_{i}=sign\left(\beta_{true}x_{i}\right)$. However, the samples might not be linearly separable, so in a general case, we consider the labeling errors, that is, some of the class labels $y_{i}$ have been flipped with probability ε. Under these assumptions, given $X=\left(x_{1},\cdots ,x_{n}\right)$, $y=\left(y_{1},\cdots ,y_{n}\right)$ and ε, the likelihood is shown in Equation (1)

(1) $$p\left(y|\beta ,\varepsilon ,X\right)=\prod_{i=1}^{n}p\left(y_{i}|\beta ,\varepsilon ,x_{i}\right)=\prod_{i=1}^{n}\left[\varepsilon \left(1-\Phi \left(y_{i}\beta^{t}x_{i}\right)\right)+\left(1-\varepsilon \right)\Phi \left(y_{i}\beta^{t}x_{i}\right)\right]$$

where Φ is the Heaviside step function and is defined by $\Phi \left(y_{i}\beta^{t}x_{i}\right)=\underset{k\to \infty }{\lim}\frac{1}{1+e^{-2k\left(y\left|\right|i\beta^{t}x_{i}\right)}}$. In fact, the likelihood function (1) is robust to outliers because it only depends on the number of errors of β in the training set and not on the actual size of these errors. In high throughput gene expression data, d≽n, indicating β can have different optimal solutions. In this study, we only considered the sparse solution for β. Herein, we introduce a new binary hidden variable $z=\left\{z_{0},z_{1},z_{2},\cdots ,z_{d}\right\}\epsilon {\left\{0,1\right\}}^{d}$. $z_{i}$ takes 0 if the $i^{th}$. component of $\beta_{true}$ is 0 and $z_{i}$ takes 1 otherwise. Assuming z is given, the prior density of β is

(2) $$p\left(\beta |z\right)=\prod_{i=1}^{d}p\left(\beta_{i}|z_{i}\right)=\prod_{i=0}^{d}[N{\left(\beta_{i},0,\sigma_{i}^{2}\right)}^{z_{i}}\left(\delta \left(\beta_{i}\right){)}^{\left(1-z_{i}\right)}\right]$$

Here, $p\left(\beta_{i}|z_{i}\right)$ is a kind of spike and slab prior, which is a mixture of a Gaussian density (the slab) and a point probability mass placed at zero (the spike). $N\left(\beta_{i},0,\sigma_{i}^{2}\right)$ represents a Gaussian density with a 0 mean and $\sigma_{i}^{2}$ variance, and $\delta \left(\beta_{i}\right)$ is an impulse function that has a probability of 1 on $\beta_{i}$ and 0 elsewhere. To complete the specification of the prior for β at zero, we assume that a network that encodes the dependencies between the gene features are known. Given a specific cancer signaling network G=(V,E) whose vertices V={0,1,⋯,d} correspond to the proteins and whose edges, E, link features that are expected to uncover the potential mechanism difference of the drug resistance samples and sensitive samples. Equation (3) shows the prior density for z given G, which is given by a Markov random field (MRF) model

(3) $$p\left(z|G,\lambda ,\gamma \right)=\frac{1}{Z}exp\left(cz_{0}+\lambda \overset{d}{\underset{i=1}{\sum }}z_{i}\right)exp\left(\gamma \sum_{\left\{u,v\right\}\in E}{\left(\frac{z_{u}}{\sqrt{d_{u}}}-\frac{z_{v}}{\sqrt{d_{v}}}\right)}^{2}\omega \left(u,v\right)\right)$$

In Equation (3), Z is a normalization constant and λ∈ℝ controls the sparsity. γ≥0 determines the sum of the square difference between $z_{u}$ and $z_{v}$ that is linked in the input network *G*, and ω(u,v) is the weight between proteins $z_{u}$ and $z_{v}$. In fact, if we assume,

$$L\left(u,v\right)=\{\begin{array}{cc} 1-\frac{w\left(u,v\right)}{d_{u}}, & if\;u=v\;and\;d_{u}\neq 0,\\ \frac{-w\left(u,v\right)}{\sqrt{d_{u}d_{v}}}, & if\;u\;and\;v\;are\;adjacent,\\ 0, & othersize. \end{array}$$

then

(4) $$p\left(z|G,\lambda ,\gamma \;\right)=\frac{1}{Z}\exp\left(cz_{0}+\;\lambda \left|z\right|\right)\exp\left(\gamma \;z^{T}Lz\;\right)$$

If the sum of square difference, ${\left(\frac{z_{u}}{\sqrt{d_{u}}}-\frac{z_{v}}{\sqrt{d_{v}}}\right)}^{2}$ is small, the subcomponent of z will be small, and a smaller solution of z will lead to a more sparsity solution of β, which will help to avoid overfitting. Furthermore, we assume the prior of ε as $p\left(\varepsilon \right)=Beta\left(\varepsilon ,a_{0},b_{0}\right)=\frac{1}{B\left(a_{0},b_{0}\right)}\varepsilon^{a_{0}-1}$, where $B\left(a_{0},b_{0}\right)$ represents the *β* function with parameters $a_{0}$ and $b_{0}$. Under the assumption above, we can use the Bayesian theorem to compute the posterior distribution of the model parameters β and ε given the training data X and y. Given the specific cancer signaling network G and the model hyper-parameters λ and γ, the posterior is given by

(5) $$p\left(\beta ,\varepsilon |y,X,G,\lambda ,\gamma \right)=\frac{\sum_{Z}p\left(y|\beta ,\varepsilon ,X\right)p\left(\beta |z\right)p\left(z|G,\lambda ,\gamma \right)p\left(\varepsilon \right)}{p\left(y|X,G,\lambda ,\gamma \right)}$$

The joint probability distributions of the model parameters and hidden variables are given as follows:

(6) p(β,ε,z,y|X,G,λ,γ)=p(y|β,ε,X)p(β|z)p(z|G,λ,γ)p(ε)

In this equation, the denominator is a normalization constant. If given a new unclassified sample $x^{test}$, we can determine its classification labels $y^{test}$ by probability as shown in Equation (7):

(7) $$p\left(y^{test}|X^{test},y,X,G,\lambda ,\gamma \right)=\iint p\left(y^{test}|\beta ,\varepsilon ,x^{test}\right)p\left(\beta ,\varepsilon |y,X,G,\lambda ,\gamma \right)d\beta d\varepsilon$$

With the Bayesian assumption above, we can easily estimate the average noise of classification labels as Eε=∬εp(β,ε|y,X,G,λ,γ)dβε. As the integrals and summations in the above three equations are difficult to calculate directly, we can make an approximate Bayesian inference for posterior probability distribution using an expectation propagation (EP) algorithm [18]. The detailed implementation of the EP algorithm for parameter estimation in NBSBM is available in the Supplementary Materials.

## 3. Results

### 3.1. Prediction of Sensitivity and Resistance of Prostate Cancer Cell Lines to Dasatinib

**Figure 1:**
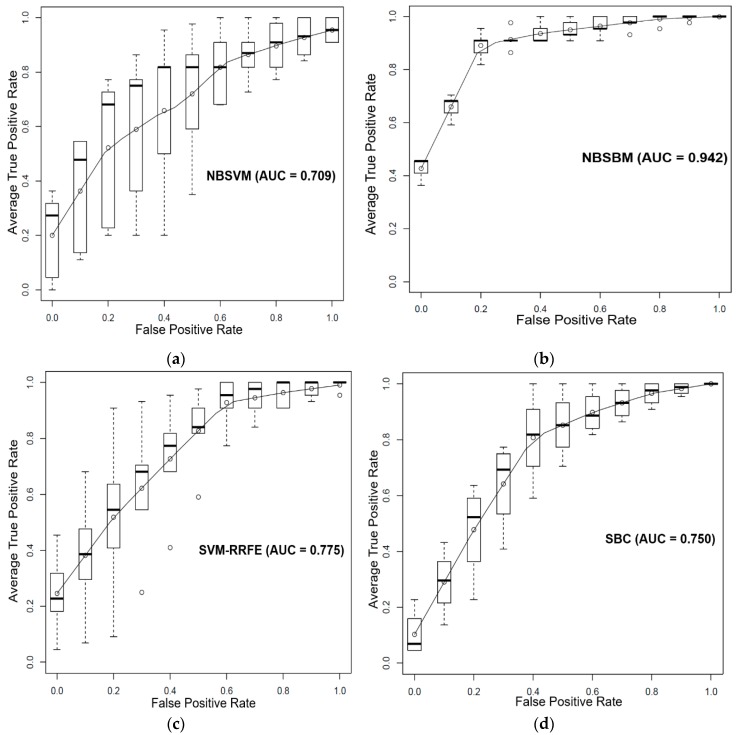
Comparison performance of the network-based sparse Bayesian machine (NBSBM) with other methods in terms of average (mean) operating characteristic (ROC) (5-fold cross-validation and 5-repeats), and AUC value. The boxplot indicates the variation around the average ROC curve and reports the median and the interquartile range. ROC curves of (**a**) network-based SVM, (**b**) the proposed approach, (**c**) SVM-RRFE, and (**d**) sparse Bayesian classifier (SBC) to classify the response of 16 prostate cancer cell lines to Dasatinib.

In Wang’s work [2], the sensitivity data of 16 prostate cancer cell lines to Dasatinib were provided. Eleven cell lines with half maximal inhibitory concentration (IC_50_) values lower than 200 nm were designated as Dasatinib-sensitive. Five cell lines with IC_50_ values larger than 200 nm were designated as Dasatinib-resistant. Previously, we reconstructed a prostate cancer-specific network [15] using multiple genomic and epigenomic data of prostate cancer patients from TCGA. There are 48 differentially expressed subnetworks (gene modules), 6738 genes, and 26,845 edges in this prostate cancer-specific network. Our goal was to predict the drug sensitivity response of these 16 prostate cell lines based on their gene expression data and the prostate cancer-specific network using the NBSBM. In this study, we set $a_{0}=1$ and $b_{0}=8$ in the NBSBM. For parameter λ(γ), we took 500 values evenly from $\left\{e^{-5},e^{2}\right\}$ ($\left\{e^{-5},e^{1}\right\}$) to select the value that achieved the lowest error rate on the training dataset. We used cross-validation (5-fold and 5-repeats) to evaluate the performance of the proposed sparse Bayesian classifier, network-based support vector machine (NBSVM) [5], support vector machine based recursive feature elimination classifier (SVM-RRFE) [19], and sparse Bayesian classifier (SBC) [13] on this dataset. We obtained the ROC curve for each algorithm by obtaining the true positive rate and average false positive rate from the cross-validation process. Figure 1 shows the ROC-curve and AUC results of the four classifiers; our method performed better than all of the other approaches. We also evaluated the differences of the predictive power of these methods by the paired Wilcoxon rank-sum test. The results show that the NBSBM achieved better results than the other two SVM-based approaches in terms of average AUC performance according to the Wilcoxon test with *p* < 0.01 (Figure 4a).

### 3.2. Prediction of Sensitivity and Resistance of Breast Cancer Patients to Tamoxifen

**Figure 2:**
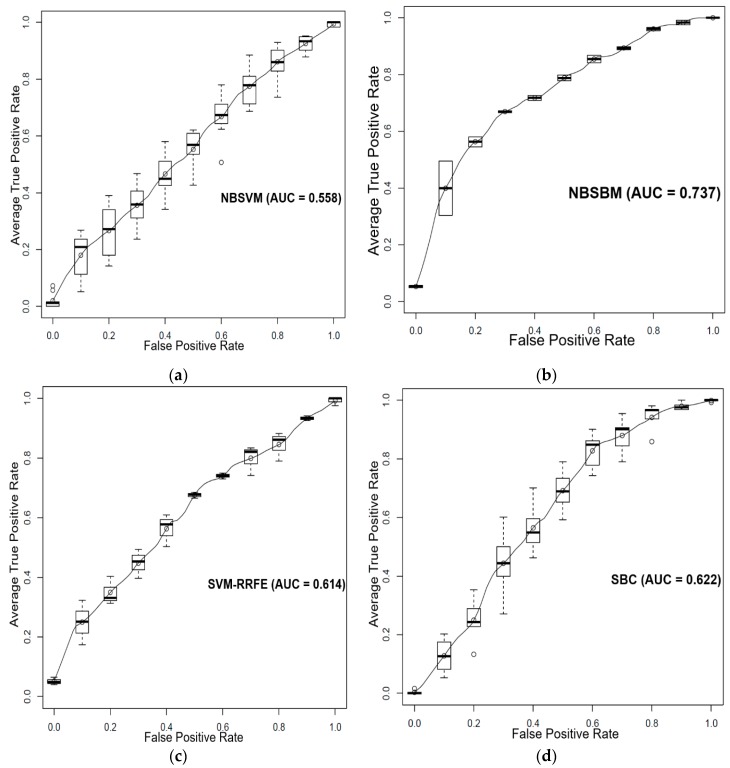
Comparison performance of the NBSBM with other methods in terms of average (mean) operating characteristic (ROC) curve (5-fold cross-validation and 5-repeats) and AUC value. The boxplot indicates the variation around the average ROC curve and reports the median and the interquartile range. ROC curves of (**a**) network-based SVM, (**b**) the proposed approach, (**c**) SVM-RRFE, and (**d**) sparse Bayesian classifier (SBC) to predict the response of estrogen receptor-positive breast cancer patients to Tamoxifen.

Dataset GSE17705 [20] (available in gene expression omnibus (GEO)) contained both the gene expression data of 103 estrogen receptor positive breast cancer patients and their survival time after Tamoxifen treatment. We divided those patients into the Tamoxifen sensitive group and Tamoxifen non-sensitive group according to their median survival time. Patients who survived longer than the median survival time were designated as Tamoxifen-sensitive, otherwise Tamoxifen-non-sensitive. Next, we employed NBSBM to predict the estrogen receptor-positive breast cancer patients’ response to Tamoxifen treatment, using a previously reconstructed estrogen receptor-positive breast cancer-specific network [16] and the gene expression data of those 103 breast cancer patients. The estrogen receptor-positive breast cancer-specific network is highly interconnected and contains 15 differentially expressed gene modules, 923 genes, and 10,073 edges. The 103 estrogen receptor positive breast cancer patients could be accurately classified by the proposed sparse Bayesian machine. We compared the proposed approach with the NBSVM and SVM-RRFE. Figure 2 shows the ROC curves and AUC results of the three classifiers individually. We found that NBSBM performed better than the other methods. We also evaluated the differences of the predictive power of these methods by the Wilcoxon rank-sum test. It can be seen that the NBSBM achieved better results than the other two-SVM approaches in terms of average AUC performance according to the Wilcoxon test with *p* < 0.05 (Figure 4b).

### 3.3. Prediction of Sensitivity and Resistance of Various Cancer Cells to Dasatinib

**Figure 3:**
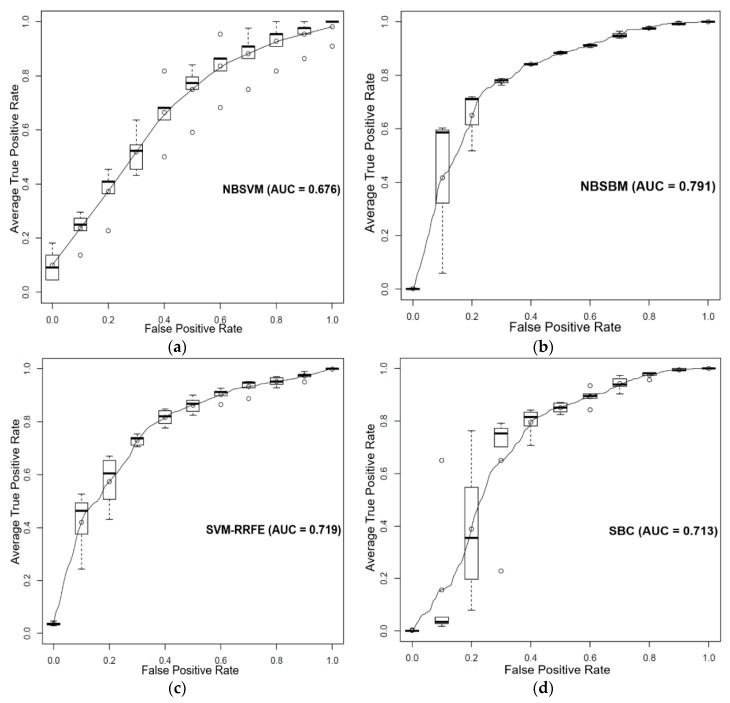
Comparison performance of the NBSBM with other methods in terms of average (mean) operating characteristic (ROC) curve (5-fold cross-validation and 5-repeats) and AUC value. The boxplot indicates the variation around the average ROC curve and reports the median and the interquartile range. ROC curves of (**a**) network-based SVM, (**b**) the proposed approach, (**c**) SVM-RRFE, and (**d**) sparse Bayesian classifier (SBC) to classify the response of estrogen receptor-positive breast cancer patients to Tamoxifen.

**Figure 4:**
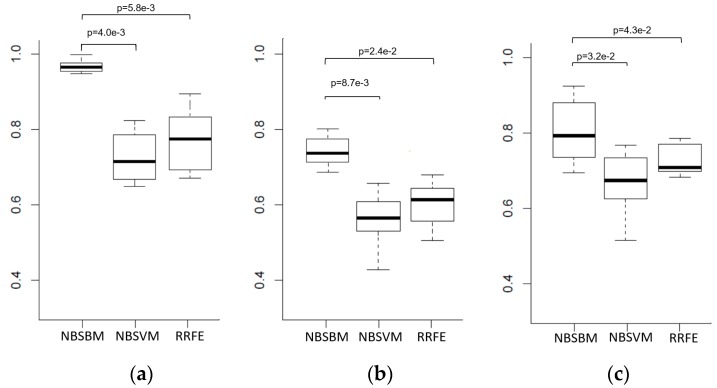
Performance comparison among NBSBM, network-based SVM, and SVM-FREE in terms of average AUC in predicting (**a**) prostate cells’ response to Dasatinib, (**b**) Breast Cancer Patients’ response to Tamoxifen therapy, and (**c**) 789 cancer cells’ response to Dasatinib. The Wilcoxon rank-sum test was used to examine whether the AUCs obtained by two approaches were different.

The Genomics of Drug Sensitivity in Cancer (GDSC) database [21,22] contains the gene expression data of 789 cancer cell lines and provides sensitivity data of various cancer cell lines to drugs from in-vitro drug screening experiments. Herein, we used the sparse Bayesian classifier to predict the sensitivity of cancer cell lines to Dasatinib based on the gene expression data of the 319 cancer cells, and an integrated cancer signaling network from our previous work [9]. The integrated human cancer signaling pathways (IHSP) consisted of previously published human cancer signaling pathways [23,24,25,26], Biocarta [27], and KEGG [28] databases. There are 7564 genes and 58,932 edges in IHSP. Figure 3 shows the ROC curve results of the three classifiers. It can be seen that our method performed better than the other two SVM algorithms in terms of average AUC performances according to the Wilcoxon rank-sum test with *p* < 0.05 (Figure 4c).

## 4. Discussion

A spike and slab prior distribution combined with a Markov-random-field (MRF) prior were used to build a spare model in the proposed network-based sparse Bayesian machine (NBSBM). Under this sparsity assumption, better results can be achieved if prior information about the gene to gene relationships with the disease-specific network is available. A disease-specific (differentially expressed) network was encoded in such prior information, in other words, MRF prior to improve the prediction performance of NBSBM. Note that the Bayesian classifier proposed in this article is capable of feature selection, in Supplementary Tables S1 and S2, we list the top relevant features (genes) and pathways that can predict prostate cancer cell responses to Dasatinib. For the top-ranked genes or pathways reported to play important roles of prostate cancer development and progression, see Supplementary Materials, Section 2 for more detail. That is, we can derive network-based biomarkers for drugs such as those highly predictive gene modules (features) from the disease-specific signaling network. Then, we can predict the sensitivity level of new cancer cells to drugs only according to the gene expression data of these network-biomarkers, which might provide an exploration of the molecular pathogenesis of specific diseases. Furthermore, those network-based biomarkers might directly contribute to drug sensitivity or resistance. In addition to the application to cancer therapeutics, our approach should be useful in predicting drug sensitivity in many common complex diseases.

## 5. Conclusions

In this article, we proposed a sparse Bayesian machine to predict the sensitivity level of cancer cells to drugs using gene expression data and disease-specific signaling networks. The Bayesian classifier systematically integrated specific cancer signaling pathways with high throughput gene expression data. It employed an expectation propagation strategy to find a sparse solution. In addition, we compared the performance of the NBSBM with other network based SVM methods. Using three different pharmacological datasets, we applied cross-validation to test the performance of the proposed Bayesian classifier. The results showed that the proposed algorithm performed much better than the other two methods, warranting further studies in individual cancer patients to predict personalized cancer treatments.

## Supplementary Materials

The following are available online at https://www.mdpi.com/2073-4425/10/8/602/s1, Table S1: Top-25 most predictive genes for classifying prostate cancer cell responses to Dasatinib; Table S2: The most enriched signaling pathways in those top-100 ranked genes that are most relevant to prostate cancer cell response to Dasatinib.
