# Supplementary material for: Network as a Biomarker: A Novel Network-Based Sparse Bayesian Machine for Pathway-Driven Drug Response Prediction
Source: Genes (Basel). 2019 Aug 9;10(8):602. doi: 10.3390/genes10080602 (PMC6723660; doi:10.3390/genes10080602)
Supplement: Supplementary file 1 [file genes-10-00602-s001.pdf]

Supplemental file

Network as a biomarker: A novel network-based sparse Bayesian machine for pathway-driven drug response prediction

Qi Liu[1,2], Louis J. Muglia[2,3] and Lei Frank Huang[1,2,4*]

1 Brain Tumor Center, Division of Experimental Hematology and Cancer Biology, Cincinnati Children's Hospital Medical Center, Cincinnati, OH 45229, USA
2 Department of Pediatrics, College of Medicine, University of Cincinnati, Cincinnati, OH 45229, USA
3 Division of Human Genetics, Cincinnati Children's Hospital Medical Center, Cincinnati, OH 45229, USA
4 Department of Information Science, School of Mathematical Sciences and LAMA, Peking University, Beijing 100871, China

* Correspondence: Frank.Huang@cchmc.org; Tel.: +15135171084

1 Approximate Bayesian Inference for parameter estimation in NBSBM using Expectation Propagation (EP) algorithm

Considering the labeling errors ε, given $\boldsymbol{X} = (\boldsymbol{x}_1, \cdots, \boldsymbol{x}_n)$, $\boldsymbol{y} = (y_1, \cdots, y_n)$ and ε, the likelihood can be written as (1)

$$p(\boldsymbol{y}|\beta, \varepsilon, \boldsymbol{X}) = \prod_{i=1}^{n} p\,(y_i|\beta, \varepsilon, x_i) = \prod_{i=1}^{n}\left[\varepsilon\left(1 - \Phi(y_i\beta^T x_i)\right) + (1-\varepsilon)\Phi(y_i\beta^T x_i)\right] = \prod_{i=1}^{n}[\varepsilon + (1-2\varepsilon)\Phi(y_i\beta^T x_i)] \quad (1)$$

Where $\boldsymbol{\Phi}$ is the Heaviside step function and it is defined by equation (2)

$$\Phi(y_i\beta^T x_i) = \lim_{k\to\infty} \frac{1}{1+e^{-2k(y_i\beta^T x_i)}} \quad (2)$$

If we only consider the sparse solution for β. Herein we introduce a new binary hidden variable $\boldsymbol{z} = \{z_0, z_1, z_2, \cdots, z_d\}\epsilon\{0,1\}^d$. z_i takes 0 if the ith component of β_{true} is 0 and z_i takes 1 otherwise. Assuming $\boldsymbol{z}$ is given, the probability density of β is shown in equation (3)

$$p(\beta|\boldsymbol{z}) = \prod_{i=1}^{d} p\,(\beta_i|z_i) = \prod_{i=0}^{d}[\mathcal{N}\left(\beta_i, 0, \sigma_i^2\right)^{z_i}(\delta(\beta_i))^{(1-z_i)}] \quad (3)$$

where $p(\beta_i|z_i)$ is a Spike and Slab prior. $\mathcal{N}(\beta_i, 0, \sigma_i^2)$ represents Gaussian density function with 0 mean and σ_i^2 variance, $\delta(\beta_i)$ is an impulse function which has a probability of 1 on β_i and 0 elsewhere. To complete the specification of the prior for $\boldsymbol{\beta}$ at zero, we assume that a network that encodes the dependencies between the gene features are known. Given a specific cancer signaling network $G = (V, E)$ whose vertices $V = \{0,1, \cdots, d\}$ correspond to the proteins and whose edges, E Equation (4) shows the prior probability for $\boldsymbol{z}$ given G which is given by a Markov random field (MRF) model

$$p(z|G,\lambda,\gamma) = \frac{1}{Z}exp\left(cz_0 + \lambda\sum_{i=1}^{d} z_i + \gamma \sum_{\{u,v\}\in E}\left(\frac{z_u}{\sqrt{d_u}} - \frac{z_v}{\sqrt{d_v}}\right)^2 w(u,v)\right) = \frac{1}{Z}exp(cz_0 + \lambda\sum_{i=1}^{d} z_i\ ) \ exp(\gamma \sum_{\{u,v\}\in E}\left(\frac{z_u}{\sqrt{d_u}} - \frac{z_v}{\sqrt{d_v}}\right)^2 w(u,v)) \tag{4}$$

In equation (2), $\boldsymbol{Z}$ *is a normalization constant and* $\lambda \in \mathbb{R}$ *controls the sparsity.* $\gamma \geq 0$ *determines the sum of square difference between* z_u *and* z_v *that are linked in the input network G,* $\omega(u,v)$ *is the weight between proteins* z_u *and* z_v*. In fact, if we assume,*

$$L(u,v) = \begin{cases} 1 - \frac{w(u,v)}{d_u}, \ if\ u = v\ and\ d_u \neq 0, \\ \frac{-w(u,v)}{\sqrt{d_u d_v}}, \ if\ u\ and\ v\ are\ adjacent, \\ 0, \qquad\qquad othersize. \end{cases} \tag{5}$$

then

$$p(\boldsymbol{z}|G,\lambda,\gamma) = \frac{1}{Z}\exp(cz_0 + \lambda|\boldsymbol{z}|)\exp(\gamma\boldsymbol{z}^T L\boldsymbol{z}\ ) \tag{6}$$

Furthermore, we assume the prior of ε *as*

$$p(\varepsilon) = Beta(\varepsilon, a_0, b_0) = \frac{1}{B(a_0,b_0)}\ \varepsilon^{a_0-1}(1-\varepsilon)^{b_0-1} \tag{7}$$

where $B(a_0,b_0)$ *represents beta function with parameters* a_0 *and* b_0*. Under the assumption above, we can use Bayesian theorem to compute the posterior distribution of the model parameters* $\boldsymbol{\beta}$ *and* ε *given the training data* $\boldsymbol{X}$ *and* $\boldsymbol{y}$*. Given the specific cancer signaling network G and the model hyper-parameters* λ *and* γ*, the posterior is given by*

$$p(\beta,\varepsilon|y,X,G,\lambda,\gamma) = \frac{\sum_Z p(y|\beta,\varepsilon,X)p(\beta|z)p(z|G,\lambda,\gamma)p(\varepsilon)}{p(y|X,G,\lambda,\gamma)} \tag{8}$$

If given a new unclassified sample x^{test}*, we can determine its classification labels* y^{test} *by probability as shown in equation (9)*

$$p(y^{test}|X^{test},y,\boldsymbol{X},G,\lambda,\gamma) = \iint p(y^{test}|\boldsymbol{\beta},\varepsilon,x^{test})p(\boldsymbol{\beta},\varepsilon|y,\boldsymbol{X},G,\lambda,\gamma)d\boldsymbol{\beta}d\varepsilon \tag{9}$$

Then the relevance of the features can be quantified by the posterior of $\boldsymbol{z}$*,*

$$p(\boldsymbol{z}|\ \boldsymbol{y},\mathbf{X},G,\lambda,\gamma) = \frac{\sum_Z\sum_\varepsilon p(\boldsymbol{y}|\boldsymbol{\beta},\varepsilon,\mathbf{X})p(\boldsymbol{\beta}|\mathbf{z})p(\mathbf{z}|G,\lambda,\gamma)p(\varepsilon)}{p(\boldsymbol{y}|\mathbf{X},G,\lambda,\gamma)} \tag{10}$$

In specific, the relevance of the i-th feature to the classification result is a value between 0 and 1 and is given by the marginal probability $p(\boldsymbol{z}|\ \boldsymbol{y},\mathbf{X},G,\lambda,\gamma)$ *with* $\boldsymbol{z} = 1$*.The higher the value, the more relevant of this gene with respect to the classification result. The joint probability distributions of model parameters and hidden variables are given as follows:*

$$p(\beta,\varepsilon,\boldsymbol{z},\boldsymbol{y}|\mathbf{X},G,\lambda,\gamma) = p(y|\beta,\varepsilon,\mathbf{X})p(\beta|\mathbf{z})p(\mathbf{z}|G,\lambda,\gamma)p(\varepsilon) \tag{11}$$

It can be written as the product of N + | E | + 3 probabilities in equation (11) according to the assumption of independence.

$$p(\beta,\varepsilon,\mathbf{z},\mathbf{y}|\mathbf{X},G,\lambda,\gamma) = [\prod_{i=1}^{n} p(y_i|\beta,\varepsilon,\mathbf{x}_i)][\prod_{i=0}^{d} p(\beta_i|z_i)]p(\mathbf{z}|G,\lambda,\gamma)p(\varepsilon) = \prod_{i=1}^{n+|E|+3} t_i(\beta,\varepsilon,\mathbf{z}) = q(\beta,\varepsilon,\mathbf{z}) \quad (12)$$

Where |E| refers to the number of edges in graph G. The first n terms of $t_i(\beta,\varepsilon,z)$ *denote the likelihood* $p(y_i|\beta,\varepsilon,x_i)$, *while* $t_{n+1}(\beta,\varepsilon,z)$, $\prod_{i=n+2}^{n+|E|+2} t_i(\beta,\varepsilon,z)$ *and* $t_{n+|E|+3}(\beta,\varepsilon,z)$ *represent* $p(\beta|\mathbf{z})$, $p(\mathbf{z}|G,\lambda,\gamma)$ *and* $p(\varepsilon)$ *respectively. According to the expectation propagation algorithm, we use* $\widetilde{t_\iota}$ *as the estimation of* t_i *and get (13)*

$$\prod_{i=1}^{n+|E|+3} t_i(\beta,\varepsilon,\mathbf{z}) \approx \prod_{i=1}^{n+|E|+3} \widetilde{t_\iota}(\beta,\varepsilon,\mathbf{z}) = Q(\beta,\varepsilon,\mathbf{z}) \quad (13)$$

It is restricted that all $\widetilde{t_\iota}$ *belong to the same exponential family of distributions, and* $Q(\beta,\varepsilon,\mathbf{z})$ *have the same expression with* $\widetilde{t_\iota}(\beta,\varepsilon,\mathbf{z})$ *because the product of functions belonging to the same exponential family of distributions is a closure. Assume that the density function of Q after normalization is* Q, *which is also the approximation of the posterior distribution* $p(\beta,\varepsilon,\boldsymbol{z},\boldsymbol{y}|\mathbf{X},G,\lambda,\gamma)$, *and use* $Q^{\setminus i}(\beta,\varepsilon,\mathbf{z})$ *to denote the approximation of* $Q(\beta,\varepsilon,z)$ *without the term* t_i *as shown in (8)*

$$Q^{\setminus i}(\beta,\varepsilon,\mathbf{z}) = \prod_{j\neq i} \widetilde{t_\iota}(\beta,\varepsilon,\mathbf{z}) = \frac{Q(\beta,\varepsilon,\mathbf{z})}{\widetilde{t_\iota}(\beta,\varepsilon,\mathbf{z})} \quad (14)$$

a general workflow of the expectation propagation algorithm for the sparse Bayesian classifier can be given as follows,

1. *Initialize all* $\widetilde{t_\iota}$ *and posterior distribution* Q;

2. *Repeat the following steps until all* $\widetilde{t_\iota}$ *converge.*

   (a) Select one $\widetilde{t_\iota}$ *that needs to be changed and calculate* $Q^{\setminus i}$: $Q^{\setminus i} = Q/\widetilde{t_\iota}$

   (b) Update the value of Q *to minimize the Kullback–Leibler(KL) divergence between* $t_iQ^{\setminus i}$ *and* $\widetilde{t_\iota}Q^{\setminus i}$.

   (c) Recalculate $\widetilde{t_\iota} = Q^{new}/Q^{\setminus i}$.

3. *Estimate model parameters.*

In fact, according to (1), (3), (4) and (5), we can approximate $\widetilde{t_\iota}$ *based on function (14)*

$$\widetilde{t_\iota}(\beta,\varepsilon,\mathbf{z}) = \widetilde{s_\iota}\varepsilon^{\widetilde{a_\iota}}(1-\varepsilon)^{\widetilde{b_\iota}} \prod_{j=0}^{d} exp(-\frac{1}{2\widetilde{v_{\iota J}}}(\beta_j - \widetilde{m_{\iota J}})^2)(z_i\widetilde{c_{\iota J}} + (1-z_i)\widetilde{d_{\iota J}}) \quad (15)$$

Where $\widetilde{\boldsymbol{m_\iota}} = (\widetilde{m_{\iota 0}}, \dots \widetilde{m_{\iota d}})^T$, $\widetilde{\boldsymbol{v_\iota}} = (\widetilde{v_{\iota 0}}, \dots \widetilde{v_{\iota d}})^T$, $\widetilde{\boldsymbol{c_\iota}} = (\widetilde{c_{\iota 0}}, \dots \widetilde{c_{\iota d}})^T$, $\widetilde{\boldsymbol{d_\iota}} = (\widetilde{d_{\iota 0}}, \dots \widetilde{d_{\iota d}})^T$ *and* $\widetilde{\boldsymbol{c_\iota}} = 1 - \widetilde{\boldsymbol{d_\iota}}$. $\widetilde{\boldsymbol{a_\iota}}$ *and* $\widetilde{\boldsymbol{b_\iota}}$ *are free parameters and* $\widetilde{s_\iota}$ *is a constant to ensure that* $\widetilde{t_\iota}Q^{\setminus i}$ *and* $t_iQ^{\setminus i}$ *get the same value when integrating. According to the previous assumption that all* $\widetilde{t_\iota}$ *belong to the same exponential family of distributions,* Q *and* $\widetilde{t_\iota}$ *have the same form and we can assume that* Q *can be expressed as shown in (15).*

$$Q(\beta,\varepsilon,\mathbf{z}) = Beta(\varepsilon|a,b)\prod_{j=0}^{d} \mathcal{N}(\beta_j|m_j,v_j)Bern(z_j|\rho_i) \quad (16)$$

Formula (15) has the same form as (16), and

$$Bern(z_i|\rho_i) \; = \; z_i\rho_i \; + \; (1-z_i)(1-\rho_i) \tag{17}$$

Where $z_i \in \{0,1\}$ *and* ρ_i *is the probability of* z_i*=1.* $\mathbf{m} = (m_0, \ldots, m_d)^T$, $\mathbf{v} = (v_0, \ldots, v)^T$, $\boldsymbol{\rho} = (\rho_0, \ldots, \rho_d)^T$. *Firstly, we initialize* Q *and* $\widetilde{t_\iota}$ *by setting* $\mathrm{a} = \mathrm{b} = 1, m_i = 0, v_i = +\infty, \rho_i = 0.5, \widetilde{m_{\iota J}} = 0, \widetilde{v_{\iota J}} = +\infty$ *and* $\widetilde{c_{\iota J}} = \widetilde{d_{\iota J}} = 1$ *for i in range [1, n+|E|+3] and j in range [0,d]. Besides, as* Q *and* $Q^{\backslash i}$ *has the same form without approximation term* $\widetilde{t_\iota}$*, we can make following assumption*

$$Q^{\backslash i}(\beta, \varepsilon, \mathbf{z}) = Beta(\varepsilon|a^{\backslash i}, b^{\backslash i}) \prod_{j=0}^{d} \mathcal{N}\left(\beta_j \middle| m_j{}^{\backslash i}, v_j{}^{\backslash i}\right) Bern(z_j|\rho_i{}^{\backslash i}) \tag{18}$$

$\boldsymbol{m}^{\backslash i} = (m_0{}^{\backslash i}, \ldots m_d{}^{\backslash i})^T$, $\boldsymbol{v}^{\backslash i} = (v_0{}^{\backslash i}, \ldots v_d{}^{\backslash i})^T$, $\boldsymbol{\rho}^{\backslash i} = (\rho_0{}^{\backslash i}, \ldots \rho_d{}^{\backslash i})^T$, $a^{\backslash i}$*and* $b^{\backslash i}$ *can be calculated based on* $Q^{\backslash i} = \; Q\; / \widetilde{t_\iota}$ *and formula (15), (16).*

$$\boldsymbol{v}^{\backslash i} = (\boldsymbol{v}^{-1} - \; \widetilde{\boldsymbol{v}}_\iota{}^{-1})^{-1} \tag{19}$$

$$\boldsymbol{m}^{\backslash i} = \boldsymbol{m} + \boldsymbol{v}^{\backslash i} \circ \widetilde{\boldsymbol{v}}_\iota{}^{-1} \circ (\boldsymbol{m} - \widetilde{\boldsymbol{m}_\iota}) \tag{20}$$

$$\boldsymbol{\rho}^{\backslash i} = \boldsymbol{\rho} \circ \widetilde{\boldsymbol{c}_\iota}^{-1} \circ (\boldsymbol{\rho} \circ \widetilde{\boldsymbol{c}_\iota}^{-1} + (1-\boldsymbol{\rho}) \circ \widetilde{\boldsymbol{d}}_\iota{}^{-1})^{-1} \tag{21}$$

$$a^{\backslash i} = a - \widetilde{a}_\iota \tag{22}$$

$$b^{\backslash i} = b - \widetilde{b}_\iota \tag{23}$$

Where $' \circ '$ *denotes the Hadamard production and the inverse of a vector means the inverse of each component of the vector. Meanwhile we have* $\widetilde{t_\iota}$ *satisfying (24)-(28) which can be used to update the value of* $\widetilde{t_\iota}$ *according to the property of the exponential family functions*

$$\mathbb{E}_{\widetilde{t_\iota}Q^{\backslash i}}[\beta] = \; \mathbb{E}_{t_i Q^{\backslash i}}[\beta] \tag{24}$$

$$\mathbb{E}_{\widetilde{t_\iota}Q^{\backslash i}}[\beta \circ \beta] = \; \mathbb{E}_{t_i Q^{\backslash i}}[\beta \circ \beta] \tag{25}$$

$$\mathbb{E}_{\widetilde{t_\iota}Q^{\backslash i}}[\mathbf{z}] = \; \mathbb{E}_{t_i Q^{\backslash i}}[\mathbf{z}] \tag{26}$$

$$\mathbb{E}_{\widetilde{t_\iota}Q^{\backslash i}}[log(\varepsilon)] = \; \mathbb{E}_{t_i Q^{\backslash i}}[log(\varepsilon)] \tag{27}$$

$$\mathbb{E}_{\widetilde{t_\iota}Q^{\backslash i}}[log(1-\varepsilon)] = \; \mathbb{E}_{t_i Q^{\backslash i}}[log(1-\varepsilon)] \tag{28}$$

We need to update the parameters in $\widetilde{t_\iota}$ *according to* $p(y_i|\beta, \varepsilon, x_i)$ *so that* $\widetilde{t_\iota}$ *match the constraints in (24)-(28) while minimizing the KL-divergence between* $\widetilde{t_\iota}Q^{\backslash i}$ *and* $t_i Q^{\backslash i}$*. We can get (29) – (30) based on (1), (18), (24), (25)*

$$\mathbb{E}_{t_i Q^{\backslash i}}[\beta] = \; \boldsymbol{m}^{\backslash i} + \; \boldsymbol{v}^{\backslash i} \nabla_m \log Z_i \tag{29}$$

$$\mathbb{E}_{t_i Q^{\backslash i}}[\beta \circ \beta] - \mathbb{E}_{t_i Q^{\backslash i}}[\beta]\mathbb{E}_{t_i Q^{\backslash i}}[\beta]^T = \; \boldsymbol{v}^{\backslash i} - \boldsymbol{v}^{\backslash i}\boldsymbol{v}^{\backslash i}(\nabla_m{}^T \nabla_m - 2\nabla_v \log Z_i) \tag{30}$$

$$Z_i = \int (\varepsilon \; + \; (1 \; - \; 2\varepsilon)\Phi(y_i \beta^T \boldsymbol{x}_i)) Beta(\varepsilon|a^{\backslash i}, b^{\backslash i}) \prod_{j=1}^{d} \mathcal{N}\left(\beta_j \middle| m_j{}^{\backslash i}, v_j{}^{\backslash i}\right) Bern(z_j|\rho_j{}^{\backslash i}) d\,\beta \tag{31}$$

By substituting (29), (30) and (31) into (24) and (25), we can get

$$\boldsymbol{m}^{new} = \boldsymbol{m}^{\setminus i} + \boldsymbol{v}^{\setminus i} \circ \frac{(1-2\bar{\varepsilon}^{\setminus i})\mathcal{N}(\lambda_i,0,1)}{\bar{\varepsilon}^{\setminus i}+(1-\bar{\varepsilon}^{\setminus i})\Phi(\lambda_i)} \frac{y_i \boldsymbol{x}_i}{\sqrt{\boldsymbol{x}_i^T \boldsymbol{v}^{\setminus i} \boldsymbol{x}_i}} \tag{32}$$

$$\boldsymbol{v}^{new} = \boldsymbol{v}^{\setminus i} - \boldsymbol{v}^{\setminus i}\boldsymbol{v}^{\setminus i}(\left(\frac{(1-2\bar{\varepsilon})\mathcal{N}(\lambda_i,0,1)}{\bar{\varepsilon}^{\setminus i}+(1-2\bar{\varepsilon}^{\setminus i})\Phi(\lambda_i)}\right)^2 \frac{(y_i\boldsymbol{x}_i)\circ(y_i\boldsymbol{x}_i)}{\boldsymbol{x}_i^T\boldsymbol{v}^{\setminus i}\boldsymbol{x}_i} - 2\frac{(1-2\bar{\varepsilon}^{\setminus i})\mathcal{N}(\lambda_i,0,1)}{\bar{\varepsilon}^{\setminus i}+(1-2\bar{\varepsilon}^{\setminus i})\Phi(\lambda_i)} - \frac{y_i(\boldsymbol{m}^{\setminus i})^T\boldsymbol{x}_i}{2} \frac{\boldsymbol{x}_i\circ\boldsymbol{x}_i}{\boldsymbol{x}_i^T\boldsymbol{v}^{\setminus i}\boldsymbol{x}_i\sqrt{\boldsymbol{x}_i^T\boldsymbol{v}^{\setminus i}\boldsymbol{x}_i}} \tag{33}$$

After simplifying (33), we get (34)

$$\boldsymbol{v}^{new} = \boldsymbol{v}^{\setminus i} - (\boldsymbol{v}^{\setminus i} \circ \boldsymbol{x}_i)(\boldsymbol{v}^{\setminus i} \circ \boldsymbol{x}_i) = \frac{y_i\alpha_i\boldsymbol{x}_i^T\boldsymbol{m}^{new}}{\boldsymbol{x}_i^T\boldsymbol{v}^{\setminus i}\boldsymbol{x}_i\sqrt{\boldsymbol{x}_i^T\boldsymbol{v}^{\setminus i}\boldsymbol{x}_i}} \tag{34}$$

In equations (32), (33) and (34), we have the following

$$\alpha_i = \frac{(1-2\bar{\varepsilon})\mathcal{N}(\lambda_i,0,1)}{\bar{\varepsilon}^{\setminus i}+(1-2\bar{\varepsilon}^{\setminus i})\Phi(\lambda_i)} \tag{35}$$

$$\lambda_i = \frac{y_i(\boldsymbol{m}^{\setminus i})^T\boldsymbol{x}_i}{\sqrt{\boldsymbol{x}_i^T\boldsymbol{v}^{\setminus i}\boldsymbol{x}_i}} \tag{36}$$

$$\bar{\varepsilon}^{\setminus i} = \frac{a^{\setminus i}}{a^{\setminus i}+ b^{\setminus i}} \tag{37}$$

$$Z_i = \bar{\varepsilon}^{\setminus i} + (1-2\bar{\varepsilon}^{\setminus i})\Phi(\lambda_i) \tag{38}$$

Here Φ is the cumulative distribution function of the standard normal distribution. According to formulas (27) and (28), we can obtain the following updating rules for a and b.

$$\Psi(a^{new}) - \Psi(a^{new}+b^{new})\frac{\bar{\varepsilon}^{\setminus i}(1-\Phi(\lambda_i))}{a^{\setminus i}[\bar{\varepsilon}^{\setminus i}+(1-2\bar{\varepsilon}^{\setminus i})\Phi(\lambda_i)]} + \Psi(a^{\setminus i}) - \Psi(a^{\setminus i}+b^{\setminus i}+1) \tag{39}$$

$$\Psi(b^{new}) - \Psi(a^{new}+b^{new})\frac{\bar{\varepsilon}^{\setminus i}(1-\Phi(\lambda_i))}{b^{\setminus i}[\bar{\varepsilon}^{\setminus i}+(1-2\bar{\varepsilon}^{\setminus i})\Phi(\lambda_i)]} + \Psi(b^{\setminus i}) - \Psi(a^{\setminus i}+b^{\setminus i}+1) \tag{40}$$

Where $\Psi(x) = dlog(\Gamma(x))$ and Γ is the gamma function. As for the fact that $\Psi(x)$ is a non-linear function, we can only use numerical solution to update a^{new} and b^{new}. In order to avoid the computational complexity, the expectation propagation of ε and ε^2 are used instead of the expectation propagation of log (ε) and log (1- ε). Although it is not guaranteed to minimize the KL divergence, the results are still accurate according to (Hernández-Lobato and Hernández-Lobato, 2008) and (Miguel Hernández-Lobato, et al., 2011). *In other words, we can use (41) and (42) to update the value of a^{new} and b^{new}*

$$\mathbb{E}_{\tilde{t}_i Q^{\setminus i}}[\varepsilon] = \mathbb{E}_{t_i Q^{\setminus i}}[\varepsilon] \tag{41}$$

$$\mathbb{E}_{\tilde{t}_i Q^{\setminus i}}[\varepsilon \circ \varepsilon] = \mathbb{E}_{t_i Q^{\setminus i}}[\varepsilon \circ \varepsilon] \tag{42}$$

After simplifying the equations we get

$$a^{new} = \frac{\mathbb{E}_{t_i Q^{\setminus i}}[\varepsilon] - \mathbb{E}_{t_i Q^{\setminus i}}[\varepsilon^2]}{\mathbb{E}_{t_i Q^{\setminus i}}[\varepsilon^2] - \mathbb{E}_{t_i Q^{\setminus i}}[\varepsilon]^2}\mathbb{E}_{t_i Q^{\setminus i}}[\varepsilon] \tag{43}$$

$$b^{new} = \frac{\mathbb{E}_{t_i Q^{\setminus i}}[\varepsilon] - \mathbb{E}_{t_i Q^{\setminus i}}[\varepsilon^2]}{\mathbb{E}_{t_i Q^{\setminus i}}[\varepsilon^2] - \mathbb{E}_{t_i Q^{\setminus i}}[\varepsilon]^2}(1 - \mathbb{E}_{t_i Q^{\setminus i}}[\varepsilon]) \tag{44}$$

In the above two equations, we have

$$\mathbb{E}_{t_i Q^{\setminus i}}[\varepsilon] = \frac{1}{Z_i(a^{\setminus i} + b^{\setminus i} + 1)}[\Phi(\lambda_i)(1 - \bar{\varepsilon}^{\setminus i})a^{\setminus i} + (1 - \Phi(\lambda_i))\bar{\varepsilon}^{\setminus i}(a^{\setminus i} + 1)] \tag{45}$$

$$\mathbb{E}_{t_i Q^{\setminus i}}[\varepsilon^2] = \frac{a^{\setminus i} + 1}{Z_i(a^{\setminus i} + b^{\setminus i} + 1)(a^{\setminus i} + b^{\setminus i} + 2)}[\Phi(\lambda_i)(1 - \bar{\varepsilon}^{\setminus i})a^{\setminus i} + (1 - \Phi(\lambda_i))\bar{\varepsilon}^{\setminus i}(a^{\setminus i} + 2)] \tag{46}$$

As for the approximation of t_{n+1}, or namely p(β|z), we have (24), (25) and (26) here according to the infer from the minimum KL divergence between $t_i Q^{\setminus i}$ and $\tilde{t}_i Q^{\setminus i}$

$$\mathbb{E}_{\tilde{t}_i Q^{\setminus i}}[\beta] = \mathbb{E}_{t_i Q^{\setminus i}}[\beta] \tag{47}$$

$$\mathbb{E}_{\tilde{t}_i Q^{\setminus i}}[\beta \circ \beta] = \mathbb{E}_{t_i Q^{\setminus i}}[\beta \circ \beta] \tag{48}$$

$$\mathbb{E}_{\tilde{t}_i Q^{\setminus i}}[\boldsymbol{z}] = \mathbb{E}_{t_i Q^{\setminus i}}[\boldsymbol{z}] \tag{49}$$

Based on the above three equations, the rules for updating ***m****,* ***v*** *and* ***ρ*** *can be derived as follows.*

$$\boldsymbol{m}^{new} = \boldsymbol{m}^{\setminus i} + k' \circ \boldsymbol{v}^{\setminus i} \tag{50}$$

$$\boldsymbol{v}^{new} = \boldsymbol{v}^{\setminus i} - k''' \circ \boldsymbol{v}^{\setminus i} \circ \boldsymbol{v}^{\setminus i} \tag{51}$$

$$\boldsymbol{\rho}^{new} = \boldsymbol{\rho}^{\setminus i} + \boldsymbol{\rho}^{\setminus i}(\boldsymbol{\rho}^{\setminus i})\nabla_{\boldsymbol{\rho}} \log Z_i \tag{52}$$

$$\boldsymbol{\rho}^{new} = \boldsymbol{\rho}^{\setminus i} + \frac{(g'' - g''')\boldsymbol{\rho}(1 - \boldsymbol{\rho}^{\setminus i})}{\boldsymbol{\rho}^{\setminus i} \circ g'' + (1 - \boldsymbol{\rho}^{\setminus i}) \circ g'''} \tag{53}$$

$$\boldsymbol{\rho}^{new} = \boldsymbol{\rho}^{\setminus i} \circ g'' \circ (\boldsymbol{\rho}^{\setminus i} \circ g'' + (1 - \boldsymbol{\rho}^{\setminus i})\, g''') \tag{54}$$

k', k''', g'' and g''' in above equations can be given as follows

$$g'' = \mathcal{N}(0, \boldsymbol{m}^{\setminus i}, \boldsymbol{v}^{\setminus i} + \sigma^2) \tag{55}$$

$$g''' = \mathcal{N}(0, \boldsymbol{m}^{\setminus i}, \boldsymbol{v}^{\setminus i}) \tag{56}$$

$$g' = \boldsymbol{\rho}^{\setminus i} \circ g'' + (1 - \boldsymbol{\rho}^{\setminus i}) \circ g''' \tag{57}$$

$$k' = -\frac{\boldsymbol{\rho}^{\setminus i} \circ g'' \circ m^{\setminus i}}{g' \circ (\boldsymbol{v}^{\setminus i} + \sigma^2)} - \frac{(1 - \boldsymbol{\rho}^{\setminus i}) \circ g''' \circ m^{\setminus i}}{g' \circ \boldsymbol{v}^{\setminus i}} \tag{58}$$

$$k'' = \frac{\boldsymbol{\rho}^{\setminus i} \circ g'' \circ m^{\setminus i} \circ m^{\setminus i}}{g' \circ (\boldsymbol{v}^{\setminus i} + \sigma^2) \circ (\boldsymbol{v}^{\setminus i} + \sigma^2)} - \frac{\boldsymbol{\rho}^{\setminus i} \circ g''}{g' \circ (\boldsymbol{v}^{\setminus i} + \sigma^2)} + \frac{(1 - \boldsymbol{\rho}^{\setminus i}) \circ g''' \circ m^{\setminus i} \circ m^{\setminus i}}{g' \circ \boldsymbol{v}^{\setminus i} \circ \boldsymbol{v}^{\setminus i}} - \frac{(1 - \boldsymbol{\rho}^{\setminus i}) \circ g'''}{g' \circ \boldsymbol{v}^{\setminus i}} \tag{59}$$

$$k''' = k' \circ k' - k'' \tag{60}$$

Z_{n+1} can be given as follows while $Beta\left(\varepsilon \middle| a^{\setminus i}, b^{\setminus i}\right)$ does not contain β and Z_i

$$Z_i = \int (\mathcal{N}(\beta_i, 0, {\sigma_i}^2)^{z_i} \delta(\beta_i)^{(1-z_i)}) \prod_{j=1}^{d} \mathcal{N}\left(\beta_j \middle| {m_j}^{\setminus i}, {v_j}^{\setminus i}\right) Bern(z_j|{\rho_j}^{\setminus i}) d\,\beta \mathrm{d}\mathbf{z} = \prod_{j=0}^{d} {g_j}' \quad (61)$$

As for the approximation of $\widetilde{t_i}$ for $t_i \epsilon \mathrm{p}(\mathbf{z}|\mathrm{G}, \lambda, \gamma)$ (i = n+2 …, n+|E|+2), we have formula (4) here

$$p(\mathbf{z}|G, \lambda, \gamma) = \frac{1}{Z} exp(cz_0) \exp(\lambda \sum_{i=1}^{d} z_i + \gamma \sum_{\{u,v\} \in E} \left(\frac{z_u}{\sqrt{d_u}} - \frac{z_v}{\sqrt{d_v}} \right)^2 w(u, v))$$

$$= \frac{1}{Z} exp(cz_0 + \lambda \sum_{i=1}^{d} z_i) \; exp(\gamma \sum_{\{u,v\} \in E} \left(\frac{z_u}{\sqrt{d_u}} - \frac{z_v}{\sqrt{d_v}} \right)^2 w(u, v)) \quad (62)$$

Firstly, we need to approximate the priori sparse term $\exp\left(cz_0 + \lambda \sum_{i=1}^{d} z_i\right)$ and the following formula holds

$$\mathbb{E}_{\widetilde{t_i} Q^{\setminus i}}[\mathbf{z}] = \mathbb{E}_{t_i Q^{\setminus i}}[\mathbf{z}] \quad (63)$$

And Z_i can be calculated by

$$Z_i = \int (\exp(h_i z_i) \, Beta(\varepsilon | a^{\setminus i}, b^{\setminus i})) \prod_{j=1}^{d} \mathcal{N}\left(\beta_j \middle| {m_j}^{\setminus i}, {v_j}^{\setminus i}\right) Bern(z_j|{\rho_j}^{\setminus i}) d\,\beta \mathrm{d}\mathbf{z} \quad (64)$$

As in the above equation, $\mathbf{h} = (h_0, h_1, \dots, h_d)^T$ is a d+1-dimension vector of which the first component is 0 while the others are λ, we can do the simplification as follows:

$$Z_i = \exp(h_i) \boldsymbol{\rho}^{\setminus i} \int \prod_{j=0}^{d} \mathcal{N}\left(\beta_j \middle| {m_j}^{\setminus i}, {v_j}^{\setminus i}\right) Bern(z_j|{\rho_j}^{\setminus i}) d\,\beta + \exp(-h_i)(1 - \boldsymbol{\rho}^{\setminus i}) \int \prod_{j=0}^{d} \mathcal{N}\left(\beta_j \middle| {m_j}^{\setminus i}, {v_j}^{\setminus i}\right) Bern(z_j|{\rho_j}^{\setminus i}) d\,\beta \quad (65)$$

$$Z_i = \prod_{j=0}^{d} [{\rho_j}^{\setminus i} \exp(h_i) + (1 - {\rho_j}^{\setminus i}) \exp(-h_i)] \quad (66)$$

According to (63), the updating rule for $\boldsymbol{\rho}$ is written as follows

$$\boldsymbol{\rho}^{new} = \boldsymbol{\rho}^{\setminus i} + \boldsymbol{\rho}^{\setminus i}(1 - \boldsymbol{\rho}^{\setminus i}) \nabla_{\boldsymbol{\rho}} \log Z_i \quad (67)$$

Combined with (66), we have

$$\boldsymbol{\rho}^{new} = \exp(\boldsymbol{h}) \circ \boldsymbol{\rho}^{\setminus i} \circ (\exp(\boldsymbol{h}) \circ \boldsymbol{\rho}^{\setminus i} + \boldsymbol{I}(1 - \boldsymbol{\rho}^{\setminus i}))^{-1} \quad (68)$$

As for the approximation of $\widetilde{t_i}$ for i in range (n+3, n+|E|+2)

$$Z_i = \int \left(\exp\left(\gamma \left(\frac{z_u}{\sqrt{d_u}} - \frac{z_v}{\sqrt{d_v}} \right)^2 \right) \right) Beta(\varepsilon | a^{\setminus i}, b^{\setminus i}) \prod_{j=1}^{d} \mathcal{N}\left(\beta_j \middle| {m_j}^{\setminus i}, {v_j}^{\setminus i}\right) Bern(z_j|{\rho_j}^{\setminus i}) d\beta d\varepsilon \quad (69)$$

Assume the A_i, B_i, C_i, D_i can be given as follows

$$A_i = \rho_u^{\setminus i} \rho_v^{\setminus i} \exp(\gamma (\frac{1}{\sqrt{d_u}} - \frac{1}{\sqrt{d_v}})^2) \quad (70)$$

$$B_i = \rho_u^{\setminus i} (1 - \rho_v^{\setminus i}) \exp(\frac{\gamma}{d_u}) \quad (71)$$

$$\mathrm{C_i} = (1-\rho_u^{\backslash i})\rho_v^{\backslash i}\exp(\frac{\gamma}{d_v}) \quad (72)$$

$$\mathrm{D_i} = (1-\rho_u^{\backslash i})(1-\rho_v^{\backslash i}) \quad (73)$$

The updating rule of ρ *can be obtained as follows*

$$\rho_u^{new} = \frac{A_i + B_i}{A_i + B_i + C_i + D_i} \quad (74)$$

$$\rho_v^{new} = \frac{A_i + C_i}{A_i + B_i + C_i + D_i} \quad (75)$$

Lastly, as for the approximation of $\widetilde{t_{n+|E|+3}}$

$$Z_i = B(a,b)B(a_0,b_0)^{-1}B(a^{\backslash i}, b^{\backslash i})^{-1} \quad (76)$$

According to the rules of propagating the expectation of ε and ε^2, we have.

$$a^{new} = a_0 + a^{\backslash i} - 1$$
$$b^{new} = b_0 + b^{\backslash i} - 1$$
$$(77)$$

We can get the following updating rules based on the above expectation propagation algorithm.

$$\widetilde{\boldsymbol{v}_\iota}^{new} = (\boldsymbol{v}^{-1} - (\boldsymbol{v}^{\backslash \boldsymbol{i}})^{-1})^{-1}$$
$$\widetilde{\mathbf{m}_\iota}^{new} = \widetilde{\boldsymbol{v}_\iota}^{new} \circ \boldsymbol{v}^{-1} \circ m - \widetilde{\boldsymbol{v}_\iota}^{new} \circ (\boldsymbol{v}^{\backslash \boldsymbol{i}})^{-1} \circ \boldsymbol{m}^{\backslash i}$$
$$\widetilde{c_\iota}^{new} = \rho \circ (\rho^{\backslash \boldsymbol{i}})^{-1}$$
$$\widetilde{d_\iota}^{new} = (1-\rho)\,(1-\rho^{\backslash \boldsymbol{i}})^{-1} \quad (78)$$
$$\widetilde{a_\iota}^{new} = a - a^{\backslash i}$$
$$\widetilde{b_\iota}^{new} = b - b^{\backslash i}$$

$\widetilde{s_\iota}$ *is a constant and its updating rule is derived from* $\widetilde{t_\iota}$*. When i = 1, 2…, n*

$$\widetilde{s_\iota}^{new} = Z_i \sqrt{\prod_{j=0}^{d} \frac{\widetilde{v_{\iota J}}^{new} + v_j^{\backslash i}}{\widetilde{v_{\iota J}}^{new}} \exp(\frac{1}{2}\sum_{j=0}^{d} \frac{(\widetilde{m_{\iota J}}^{new} - m_j^{\backslash i})^2}{\widetilde{v_{\iota J}}^{new} + v_j^{\backslash i}})\frac{B(a^{\backslash i}, b^{\backslash i})}{B(a,b)}} \quad (79)$$

When i = $n+2, \dots, n+|E|+2$*, the updating rule of* $\widetilde{s_\iota}$ *becomes*

$$\widetilde{s_\iota}^{new} = Z_i \prod_{j=0}^{d} \sqrt{\frac{\widetilde{v_{\iota J}}^{new} + v_j^{\backslash i}}{\widetilde{v_{\iota J}}^{new}}} \exp(\frac{1}{2}\frac{(k_j')^2}{k_j'''}) \quad (80)$$

When i = $n+2, \dots, n+|E|+2$*, the updating rule of* $\widetilde{s_\iota}$ *becomes*

$$\widetilde{s_\iota}^{new} = Z_i \quad (81)$$

When i = $n + |E| + 3$, the updating rule becomes

$$\widetilde{s_i}^{new} = B(a_0, b_0)^{-1} \quad (82)$$

Once the expectation propagation algorithm converges, we can approximate it according to the following formula

$$p(y|\mathbf{x}, G, \lambda, \gamma) \approx \int \sum_z \prod_{i=1}^{n+|E|+3} \widetilde{t_i}(\beta, \varepsilon, z)\, d\beta d\varepsilon \approx \hat{Z}^{-1} C(2\Pi)^{\frac{d}{2}} \exp(\frac{D}{2}) B(A, B)[\prod_{i=1}^{n+|E|+3} \widetilde{s_i}][\prod_{j=0}^{d} \sqrt{v_j}] \quad (83)$$

Where as

$$A = \sum_{i=1}^{n+|E|+3} \widetilde{a_i} + 1 \quad (84)$$

$$B = \sum_{i=1}^{n+|E|+3} \widetilde{b_i} + 1 \quad (85)$$

$$C = \prod_{j=0}^{d} (\prod_{i=1}^{n+|E|+3} \widetilde{c_{ij}} + \prod_{i=1}^{n+|E|+3} \widetilde{d_{ij}}) \quad (86)$$

$$D = \boldsymbol{m}^T(\boldsymbol{v}^{-1} \circ \boldsymbol{m}) - \sum_{i=1}^{n+|E|+3} \widetilde{\boldsymbol{m}_i}^T (\widetilde{v_i}^{-1} \circ \widetilde{\boldsymbol{m}_i}) \quad (87)$$

$\hat{Z}$ is the approximation of Z in (3). Finally, we can predict the label of new samples according to the following formula

$$p(y^{test}|x^{test}, y, \boldsymbol{X}, G, \lambda, \gamma) \approx \int\int p(y^{test}|x^{test}, \beta, \varepsilon, G, \lambda, \gamma) \sum_z p(\beta, \varepsilon, z|y, \boldsymbol{X}, G, \lambda, \gamma)\, d\beta d\varepsilon = \int\int [\varepsilon + (1 - 2\varepsilon)\phi(y^{test}\beta x^{test})] \sum_z \mathcal{Q}(\beta, \varepsilon, z)\, d\beta d\varepsilon \quad (88)$$

According to (14)

$$p(y^{test}|x^{test}, y, \boldsymbol{X}, G, \lambda, \gamma) \approx \int\int [\varepsilon + (1 - 2\varepsilon)\phi(y^{test}\beta x^{test})] \sum_z Beta(\varepsilon|a, b) \prod_{j=0}^{d} \mathcal{N}(\beta_j|m_j, v_j) Bern(z_j|\rho_i)\, d\beta d\varepsilon \quad (89)$$

After simplification, we have

$$p(y^{test}|x^{test}, y, \boldsymbol{X}, G, \lambda, \gamma) \approx \bar{\varepsilon} + (1 - 2\bar{\varepsilon})\phi\left(\frac{y^{test}\boldsymbol{m}^T\boldsymbol{x}^{test}}{\sqrt{(\boldsymbol{v} \circ \boldsymbol{x}^{test})^T \boldsymbol{x}^{test}}}\right) \quad (90)$$

Where as

$$\bar{\varepsilon} = \frac{a}{a+b} \quad (91)$$

2 Feature selection in NBSBM

A relevant score was defined by equation (10) to quantify the relevance of a feature to the classification results. We applied equation (10) on the first dataset to extract features that are most relevant to the prostate cancer cell responses to Dasatinib. Supplementary table 1 shows those top-25 relevant genes that ranked by the relevant score. Among the top-ranked genes, CTNNB1, FGFR4, GRK6 and PHB2 are oncogenes that have been reported to play important role in prostate cancer development and progression (FitzGerald, et al., 2009; Linch, et al., 2017; Nakai, et al., 2019; Yang, et al., 2018). Then we did canonical

pathway enrichment analysis, those significantly enriched pathways were listed out in Supplementary table 2. MHC class II antigen presentation, Integration of energy metabolism, MAPK family signaling cascades, RAF/MAP kinase cascade, FLT3 Signaling pathways are top-enriched signaling pathways that correlated with the prostate cancer cell responses to Dasatinib, which was also reported by the literature(da Silva, et al., 2013; Mukherjee, et al., 2011; Younger, et al., 2007).

Gene Entrez ID	Gene Symbol	Relevant Score
**1499**	CTNNB1	0.9999
**51005**	AMDHD2	0.9999
**2264**	FGFR4	0.9998
**2870**	GRK6	0.9913
**11331**	PHB2	0.9913
**8504**	PEX3	0.9913
**8851**	CDK5R1	0.9913
**80700**	UBXN6	0.9913
**8078**	USP5	0.9913
**9409**	PEX16	0.9913
**22826**	DNAJC8	0.9913
**7317**	UBA1	0.9913
**55968**	NSFL1C	0.9913
**3053**	SERPIND1	0.9913
**57591**	MRTFA	0.9913
**10635**	RAD51AP1	0.9913
**8541**	PPFIA3	0.9913
**4601**	MXI1	0.9913
**55844**	PPP2R2D	0.9913
**5526**	PPP2R5B	0.9913
**51400**	PPME1	0.9913
**3009**	H1-5	0.9913
**9989**	PPP4R1	0.9913
**57718**	PPP4R4	0.9913

Supplemental Table 1 Top-25 most predictive genes for classifying prostate cancer cell responses to Dasatinib. Oncogenes such as CTNNB1, FGFR4, GRK6 and PHB2 are top-ranked.

Enriched Pathways	p-value
**MHC class II antigen presentation**	2.74E-06
**Integration of energy metabolism**	0.002008
**MAPK family signaling cascades**	0.002191
**RAF/MAP kinase cascade**	0.006537
**FLT3 Signaling**	0.006955
**MAPK1/MAPK3 signaling**	0.009353
**interleukin signaling**	0.012823

**Rho GTPase cycle**	0.015637
**Downstream TCR signaling**	0.027074
**Signaling by Receptor Tyrosine Kinases**	0.028316
**RHO GTPases Activate Formins**	0.037878

Supplemental Table 2 The most enriched signaling pathways in those top-100 ranked genes that are most relevant to prostate cancer cell response to Dasatinib. P-value was estimated using the fisher's exact test.

References

da Silva, H.B.*, et al.* (2013) Dissecting Major Signaling Pathways throughout the Development of Prostate Cancer, *Prostate Cancer*, **2013**, 23.

FitzGerald, L.M.*, et al.* (2009) Association of FGFR4 genetic polymorphisms with prostate cancer risk and prognosis, *Prostate Cancer Prostatic Dis*, **12**, 192-197.

Hernández-Lobato, D. and Hernández-Lobato, J.M. (2008) Bayes Machines for binary classification, *Pattern Recognition Letters*, **29**, 1466-1473.

Linch, M.*, et al.* (2017) Intratumoural evolutionary landscape of high-risk prostate cancer: the PROGENY study of genomic and immune parameters, *Annals of oncology : official journal of the European Society for Medical Oncology*, **28**, 2472-2480.

Miguel Hernández-Lobato, J., Hernández-Lobato, D. and Suárez, A. (2011) Network-based sparse Bayesian classification, *Pattern Recognition*, **44**, 886-900.

Mukherjee, R.*, et al.* (2011) Upregulation of MAPK pathway is associated with survival in castrate-resistant prostate cancer, *British journal of cancer*, **104**, 1920-1928.

Nakai, A.*, et al.* (2019) The COMMD3/8 complex determines GRK6 specificity for chemoattractant receptors, *The Journal of Experimental Medicine*, **216**, 1630-1647.

Yang, J., Li, B. and He, Q.-Y. (2018) Significance of prohibitin domain family in tumorigenesis and its implication in cancer diagnosis and treatment, *Cell Death Dis*, **9**, 580-580.

Younger, A.R.*, et al.* (2007) HLA class II antigen presentation by prostate cancer cells, *Prostate Cancer Prostatic Dis*, **11**, 334.
